# Ethnicity and anthropometric deficits in children: A cross-sectional analysis of national survey data from 18 countries in sub-Saharan Africa

**DOI:** 10.1371/journal.pgph.0003067

**Published:** 2024-12-31

**Authors:** Lucy S. Tusting, Swapnil Mishra, Harry S. Gibson, Steven W. Lindsay, Daniel J. Weiss, Seth Flaxman, Samir Bhatt

**Affiliations:** 1 Department of Disease Control, London School of Hygiene & Tropical Medicine, London, United Kingdom; 2 Saw Swee Hock School of Public Health and Institute of Data Science, National University of Singapore and National University Hospital, Singapore, Singapore; 3 Nuffield Department of Medicine, Big Data Institute, University of Oxford, Oxford, United Kingdom; 4 Department of Biosciences, Durham University, Durham, United Kingdom; 5 Telethon Kids Institute, Perth, Australia; 6 Curtin University, Perth, Australia; 7 Department of Computer Science, University of Oxford, Oxford, United Kingdom; 8 Section of Epidemiology, Department of Public Health, University of Copenhagen, Copenhagen, Denmark; 9 Department of Infectious Disease Epidemiology, Imperial College London, London, United Kingdom; PLOS: Public Library of Science, UNITED STATES OF AMERICA

## Abstract

Child anthropometric deficits remain a major public health problem in Sub-Saharan Africa (SSA) and are a key target of the UN Sustainable Development Goals (SDGs). The SDGs recommend disaggregation of health indicators by ethnic group. However, few studies have assessed how ethnicity is associated with anthropometric deficits across SSA. Data were extracted from 37 georeferenced Demographic and Health Surveys carried out during 2006–2019 across SSA that recorded anthropometric data for children aged <5 years. In a cross-sectional analysis, the odds of stunting (low height-for-age), wasting (low weight-for-height) and underweight (low weight-for-age) were modelled in relation to ethnic group using a generalised linear hierarchical mixed-effects model, controlling for survey design and environmental, socioeconomic and clinical variables. The study population comprised 138,312 children spanning 45 ethnic groups across 18 countries. In pairwise comparisons (accounting for multiple comparisons) between ethnic groups, height-for-age z-scores differed by at least 0.5 standard deviations in 29% of comparisons, weight-for-height z-scores in 36% of comparisons and weight-for-age z-scores in 20% of comparisons. Compared to a reference group of Fula children (the largest ethnic group), ethnic group membership was associated with both increases and decreases in growth faltering, ranging from a 69% reduction to a 32% increase in odds of stunting (Igbo: adjusted odds ratio (aOR) 0.31, 95% confidence intervals (CI) 0.27–0.35, p<0.0001; Hausa: aOR 1.32, 95% CI 1.21–1.44, p<0.0001); a 13% to 87% reduction in odds of wasting (Mandinka: aOR 0.87, 95% CI 0.76–0.99, p = 0.034; Bamileke: aOR 0.13, 95% CI 0.05–0.32, p<0.0001) and an 85% reduction to 13% increase in odds of underweight (Bamileke: aOR 0.15, 95% CI 0.08–0.29, p<0.0001; Hausa: aOR 1.13, 95% CI 1.03–1.24, p = 0.010). Major ethnic disparities in stunting, wasting and underweight were observed across 18 countries in SSA. Understanding and accounting for these differences is essential to support progress monitoring and targeting of nutrition interventions in children.

## Introduction

Child anthropometric deficits remain a major public health problem in sub-Saharan Africa (SSA) and are a key target of the UN Sustainable Development Goals (SDGs). The Global Nutrition Targets, incorporated into the 2030 SDGs, aim to reduce by 2025 the number of stunted children aged <5 years by 40% and the prevalence of early childhood wasting to below 5% [1]. While child growth has improved in many populations, fewer than half of low- and middle-income countries (LMICs) are expected to reach this goal [2]. In SSA, poor growth outcomes present a major public health challenge [3] with an estimated 37% of children aged <5 years stunted (insufficient height-for-age), 9% wasted (insufficient weight-for-height) and 20% underweight (insufficient weight-for-age) in 2015 [2]. These deficits exacerbate other primary causes of childhood morbidity and mortality in SSA, ultimately accounting for an estimated 23% of deaths of children aged <5 years in this region in 2015 [4].

Quantitative measurements of trends and distribution of disease burden are necessary to measure progress towards the SDGs and improve intervention targeting. For indicators of children’s growth including stunting, wasting and underweight, high-resolution estimates of national [4] and sub-national [2] disease burden have been produced. However, the SDGs also advocate for examination of progress towards individual goals disaggregated by socioeconomic and demographic variables. Ethnicity, which defines a large population group with a shared culture, language and heritage, is a key factor associated with health risks and outcomes [5]. Child growth outcomes have been investigated relative to ethnicity in two multi-country studies [6, 7] and compared between populations in pooled international analyses [8–10]. Growth outcomes have also been measured in multi-country, population-based cohorts designed to support the development of international growth standards (including the World Health Organization (WHO) Multicentre Growth Reference Study (MGRS) [11] and the INTERGROWTH-21^st^ study [12]); however, these studies did not specifically investigate inequalities between ethnic groups. To our knowledge, no studies have systematically measured the association between ethnicity and anthropometric deficits across SSA. Here we investigated whether there are significant differences in anthropometric deficits among children aged <5 years belonging to different ethnic groups across SSA using nationally representative survey data, adjusting for environmental, socioeconomic and clinical covariables.

## Materials and methods

### Data sources

**Fig 1:**
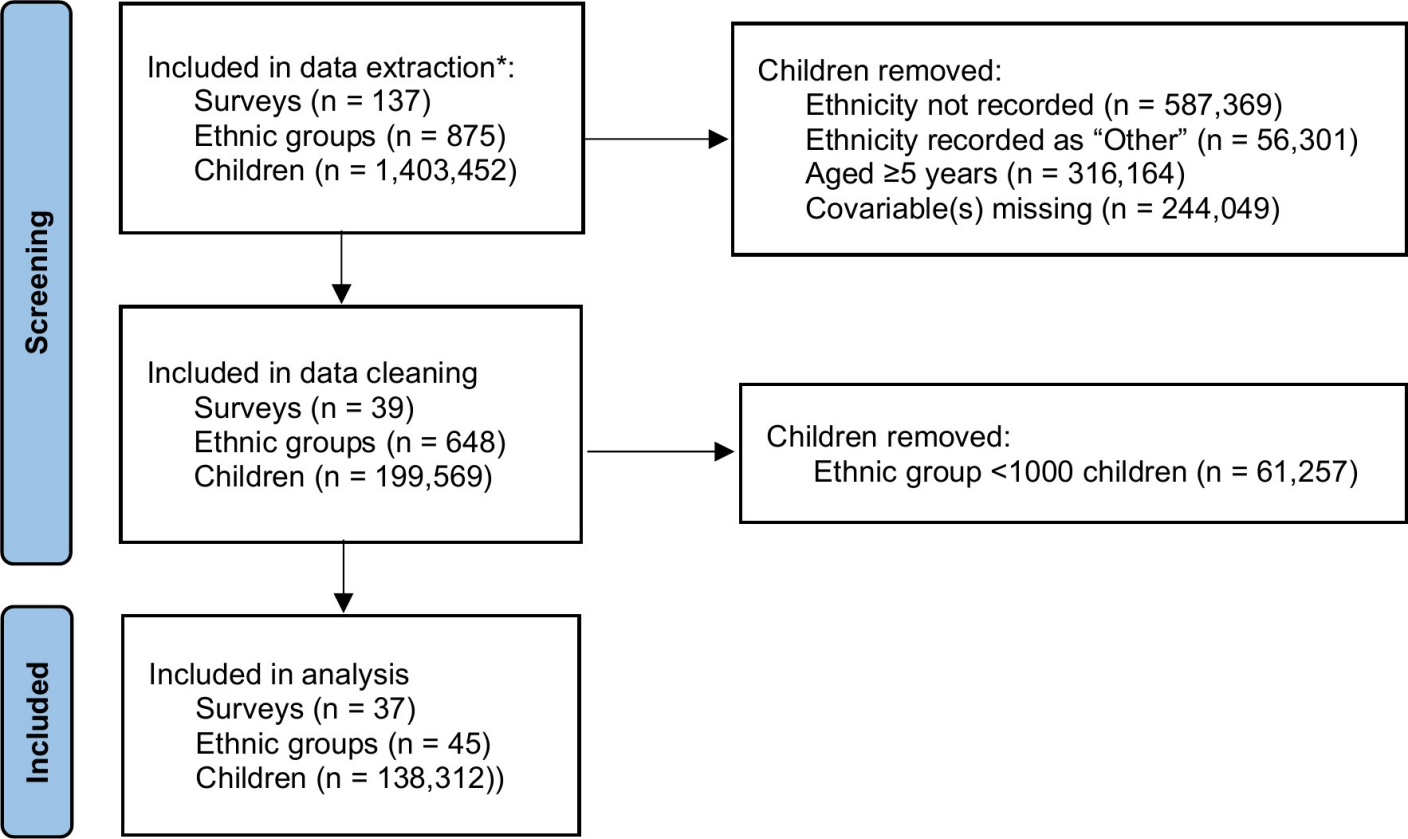
Study flow. All georeferenced surveys from sub-Saharan Africa that were published by February 2021 and collected anthropometric data and all prespecified covariables were included in the data extraction.

**Fig 2:**
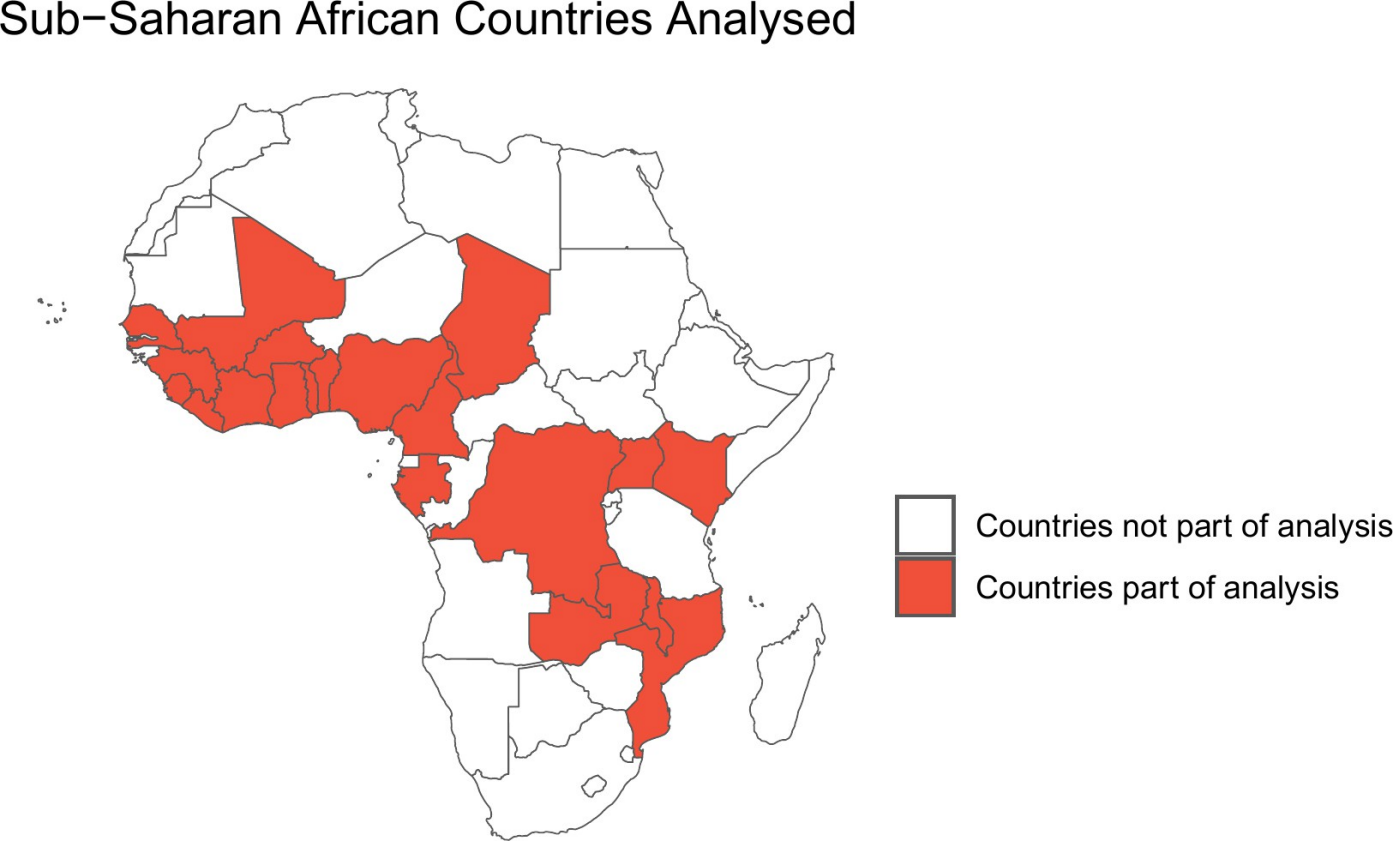
Countries included in the analysis. The map was generated with data from Natural Earth using the R package "rnaturalearth" (v0.3.2) (https://github.com/ropensci/rnaturalearth).

Data were extracted from the Demographic and Health Survey (DHS) program. DHS surveys are conducted every 3–5 years using a two-stage random cluster sampling strategy to collect nationally representative health and sociodemographic data [13]. We included any georeferenced surveys conducted in SSA that were published by February 2021 and that collected anthropometric data and all prespecified covariables (Figs 1 and 2). All available surveys for individual countries were included to maximise the study population. Data were accessed for research purposes on 20^th^ February 2021 and the study authors had no access to information that could identify individual participants during or after data collection.

#### Growth outcomes

Anthropometric data were extracted for children aged <5 years. Stunting was defined as a height-for-age z-score (HAZ) greater than two standard deviations (SD) below the WHO 2006 reference median, wasting as a weight-for-height z-score (WHZ) lower than -2 SD, and underweight as a weight-for-age z-score (WAZ) lower than -2 SD. The z-scores were calculated using the WHO *anthro* package in R. WHO 2006 criteria for cleaning z-scores were applied, where children were excluded if they had HAZ scores below -6 SD or above +6 SD, WAZ scores below -6 SD or above +5 SD, or WHZ scores below -5 SD or above +5 SD [14]. Like WHO, we used 0.5 SD as a benchmark for clinically significant differences [11].

#### Ethnicity

Within survey households, the mother or primary caregiver of each child provided self-reported ethnicity. The named ethnicity was assumed to apply to each child. Ethnic groups with fewer than 1,000 children were excluded to prevent spurious relationships from low sample sizes and to limit the overall number of ethnic groups. The largest ethnic group (Fula) was used as the reference group to prevent a rank deficient design matrix.

#### Child and household characteristics

The following individual-level covariables were extracted for each child: age, gender, reported use of an insecticide-treated net (ITN) the previous night, receipt of the third diphtheria-pertussis-tetanus (DPT-3) vaccination, receipt of the first measles vaccination (measles-1), any reported episode of diarrhoea in the previous two weeks, whether or not the child was exclusively breastfed (for children aged <1 year), and whether height was measured standing or lying. Child-level covariables were linked with the following household-level covariables: improved or unimproved drinking water source and sanitation facility categorised using WHO Joint Monitoring Programme criteria [15] (i.e. for drinking water, whether or not the source has adequate protection from outside contamination–improved sources include piped water, protected wells and rainwater; for sanitation facility, whether or not the facility adequately separates human excreta from human contact), education level of the household head, urban or rural residence, and finished *versus* unfinished or natural floor. Since household wealth scores were missing for some surveys, a household wealth index was re-calculated across all surveys using linear principal component analysis using methods described elsewhere [16]. The following assets were included: bicycle, car, cart, electricity, landline telephone, mobile telephone, motorboat, radio, refrigerator, scooter, television and watch. However, for each survey we excluded assets with more than 10% missing values and a population frequency <5% or >95%.

#### Climate and environmental variables

For each survey cluster the following data were extracted: synoptic mean monthly daytime land surface temperature (LST) from 2000 to 2016 (i.e. the average temperature for each month derived from a multiyear time series) [17]; synoptic mean monthly enhanced vegetation index (EVI) from 2000 to 2020, which is correlated with vegetation density [17]; synoptic total monthly rainfall from 2000 to 2020 [18] and accessibility to large cities in 2015 [19].

#### Association between stunting and ethnicity

The association between ethnicity and six growth outcomes (stunting, wasting, underweight, HAZ score, WHZ score and WAZ score) was modelled using a generalised linear hierarchical mixed effects model. The model included a fixed effects design matrix (*X*) for age and gender of the child, ITN use, DPT-3 vaccination, measles-1 vaccination, reported diarrhoea, height measurement position, drinking water source, sanitation facility, secondary education of the household head, urban or rural survey cluster, household floor material, household wealth, monthly mean LST, monthly mean EVI, mean total monthly rainfall and accessibility to large cities. Breastfeeding status was not included in the model due to high missing data. Except for age, continuous fixed effects were standardised to make them directly interpretable with binary fixed effects. The linear predictor of z-scores and binary growth outcomes for a given child *i* was modelled as:

$$\mu_{i}=X_{i}\beta^{T}+U_{i,s}+V_{i,s,c}$$

Where the terms *U_i,s_* are survey random effects, *V_i,s,c_* are survey-cluster random effects and *X_i_* is the design matrix of covariables. Conceptually, the covariables account for the effect of wealth, education and other factors, the survey effects provide an adjustment factor based on the survey and the survey-cluster effects provide a further adjustment to a specific cluster within a given survey. A normal likelihood was used for fitting z-scores and a binomial likelihood for binary growth outcomes. Fitting was performed using generalized linear mixed models using Template Model Builder *via* restricted maximum likelihood [20]. Standard errors and confidence intervals were estimated using the Wald method. Pairwise comparisons to compute a linear hypothesis difference in means was performed *via* Tukeys method, adjusting for multiple comparisons. Model performance is described in S1 Text.

#### Role of the funding source

The study sponsors had no role in the study design; in the collection, analysis, and interpretation of data; in the writing of the report; or in the decision to submit the paper for publication.

#### Ethics

Procedures and questionnaires for DHS surveys were reviewed and approved by the ICF Institutional Review Board (IRB). Additionally, country-specific DHS survey protocols were reviewed by the ICF IRB and typically by an IRB in the host country. Prior to each interview, informed consent was obtained for all participants, or from a parent or guardian prior to participation by a child or adolescent. The informed consent statement emphasised that participation is voluntary; that the respondent may refuse to answer any question, decline any biomarker test, or terminate participation at any time; and that the respondent’s identity and information would be kept confidential.

## Results

### Study population

Descriptive statistics are shown in S1 and S2 Tables. Data were extracted for 137 AIDS, DHS, MIS and SPE surveys in Asia, Latin America and SSA, of which 37 DHS surveys that measured all pre-specified variables were analysed (Figs 1 and 2). The included surveys were conducted during 2006–2019 in 18 countries, all of which were in SSA. A total of 138,312 children had complete data and belonged to an ethnic group with at least 1,000 observations. These children were resident in 92,588 households spanning 45 ethnic groups, the largest being Fula (n = 17,558 children, 12.7%). Median cluster size was 10 households (interquartile range 6, 14). The mean age of children was 2.2 years (95% confidence intervals (CI) 2.2, 2.2) and 69,478 (50.2%) children were male.

### Association between stunting and ethnicity

**Fig 3:**
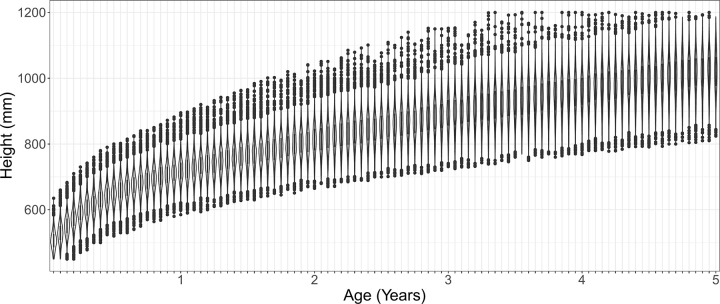
Variation in height-for-age by ethnicity. Points represent median of absolute height-for-age for each ethnic group.

**Fig 4:**
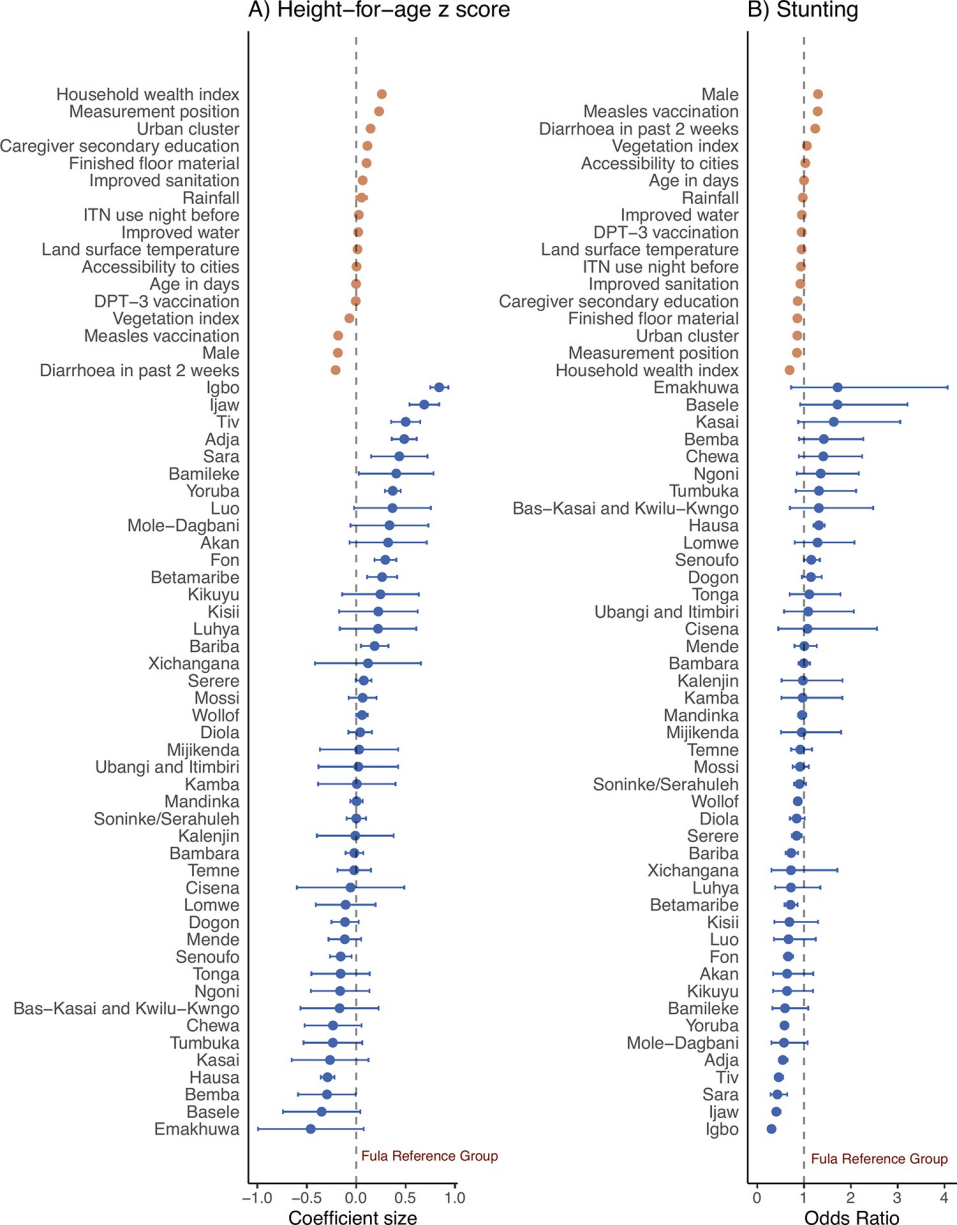
Association between ethnicity and (a) height-for-age *z* (HAZ) score and (b) stunting among children aged <5 years in 18 countries in sub-Saharan Africa surveyed between 2006 to 2019. Effect estimates and 95% confidence intervals (CI) are from a generalised linear hierarchical mixed effects model controlling for all variables shown. Stunting is defined as a HAZ score more than two standard deviations lower than the World Health Organisation reference median [36].

Overall, 46,135 (33.4%) of 138,312 children were stunted and 81% had a height-for-age lower than the WHO median. Stunting prevalence ranged from 17.8% (1,026 of 5,752) in Igbo children to 52.3% (6,555 of 12,540) in Hausa children (Tables 1 and 2). Greater variation in HAZ scores was found between, than within, ethnic groups (p<0.0001) (Fig 3). HAZ scores differed by at least 0.5 SD in 29% of significant pairwise comparisons between ethnic groups. The largest difference between any two ethnic groups was 1.30 SD. Compared to Fula children, the adjusted difference in HAZ score associated with ethnicity ranged from 0.84 SD higher (95% CI 0.75–0.93, p<0.0001) in Igbo children to 0.30 SD lower (95% CI -0.59, -0.00, p = 0.047) in Bemba children. Compared to Fula children, the adjusted difference in odds of stunting associated with ethnicity ranged from 69% lower odds in Igbo children (adjusted Odds Ratio (OR) 0.31, 95% CI 0.27–0.35, p<0.0001) to 32% higher odds in Hausa children (aOR 1.32, 95% CI 1.21–1.44, p<0.0001) (Fig 4 and S3 Table). As a comparison, the association between stunting and other covariables ranged from 31% lower odds of stunting in wealthier compared to less wealthy households (aOR 0.69, 95% CI 0.67–0.72; p<0.0001) to 30% higher odds of stunting in boys compared to girls (aOR 1.30, 95% CI 1.27–1.33, p<0.0001).

**Table 1:** Anthropometric characteristics of surveys included in the analysis (n = 37).

DHS Survey	N	Height in cm (median, N)	HAZ score (median, N)	Stunted (%, N)	WHZ score (median, N)	Wasted (%, N)	WAZ score (median, N)	Underweight (%, N)
Benin 2012	5589	83.0 (5589)	-1.62 (5589)	42.5 (5589)	-0.02 (5589)	15.8 (5589)	-0.93 (5589)	20.2 (5589)
Benin 2017	5975	75.4 (5975)	-1.37 (5975)	30.8 (5975)	-0.30 (5975)	6.3 (5975)	-0.98 (5975)	17.3 (5975)
Burkina Faso 2010	3873	83.0 (3873)	-1.41 (3873)	33.7 (3873)	-0.63 (3873)	17.0 (3873)	-1.21 (3873)	25.4 (3873)
Cameroon 2011	926	84.2 (926)	-0.99 (926)	22.5 (926)	0.78 (926)	1.2 (926)	0.04 (926)	2.9 (926)
Cameroon 2018	245	78.2 (245)	-0.70 (245)	18.0 (245)	0.99 (245)	1.2 (245)	0.38 (245)	3.7 (245)
Chad 2014	1886	83.0 (1886)	-1.13 (1886)	29.7 (1886)	-0.17 (1886)	9.6 (1886)	-0.78 (1886)	17.7 (1886)
DRC 2007	2099	82.0 (2099)	-1.78 (2099)	44.9 (2099)	-0.13 (2099)	9.5 (2099)	-1.00 (2099)	22.0 (2099)
DRC 2013	5479	81.9 (5479)	-1.79 (5479)	45.6 (5479)	-0.14 (5479)	7.6 (5479)	-1.10 (5479)	23.7 (5479)
Cote d’Ivoire 2012	644	81.6 (644)	-1.22 (644)	29.5 (644)	-0.23 (644)	5.6 (644)	-0.88 (644)	13.0 (644)
Ghana 2008	1385	84.2 (1385)	-1.21 (1385)	28.7 (1385)	-0.37 (1385)	9.1 (1385)	-0.89 (1385)	14.8 (1385)
Ghana 2014	1707	84.5 (1707)	-0.96 (1707)	18.6 (1707)	-0.30 (1707)	5.5 (1707)	-0.75 (1707)	11.4 (1707)
Guinea 2012	2150	83.0 (2150)	-1.22 (2150)	31.4 (2150)	-0.41 (2150)	11.5 (2150)	-0.91 (2150)	19.3 (2150)
Guinea 2018	1416	75.3 (1416)	-1.03 (1416)	27.6 (1416)	-0.34 (1416)	10.1 (1416)	-0.81 (1416)	17.1 (1416)
Kenya 2008	3696	83.5 (3696)	-1.46 (3696)	34.7 (3696)	0.01 (3696)	5.8 (3696)	-0.78 (3696)	14.8 (3696)
Kenya 2014	12068	85.0 (12068)	-1.25 (12068)	27.5 (12068)	0.06 (12068)	3.3 (12068)	-0.66 (12068)	10.6 (12068)
Liberia 2013	118	85.2 (118)	-1.32 (118)	26.3 (118)	-0.29 (118)	4.2 (118)	-0.92 (118)	11.0 (118)
Malawi 2010	3065	83.0 (3065)	-1.88 (3065)	46.6 (3065)	0.34 (3065)	3.8 (3065)	-0.75 (3065)	12.3 (3065)
Malawi 2015	2267	76.5 (2267)	-1.52 (2267)	34.7 (2267)	0.15 (2267)	3.5 (2267)	-0.70 (2267)	10.8 (2267)
Mali 2006	5339	80.0 (5339)	-1.40 (5339)	35.7 (5339)	-0.56 (5339)	16.3 (5339)	-1.16 (5339)	25.6 (5339)
Mali 2012	3612	84.3 (3612)	-1.47 (3612)	38.3 (3612)	-0.47 (3612)	12.8 (3612)	-1.16 (3612)	25.9 (3612)
Mali 2018	570	76.8 (570)	-1.04 (570)	24.9 (570)	-0.55 (570)	12.3 (570)	-0.96 (570)	20.2 (570)
Mozambique 2011	4219	82.3 (4219)	-1.67 (4219)	39.6 (4219)	0.27 (4219)	5.0 (4219)	-0.76 (4219)	14.3 (4219)
Nigeria 2008	10722	82.2 (10722)	-1.54 (10722)	40.4 (10722)	-0.18 (10722)	15.9 (10722)	-1.02 (10722)	24.9 (10722)
Nigeria 2013	15458	82.4 (15458)	-1.43 (15458)	38.1 (15458)	-0.56 (15458)	17.9 (15458)	-1.24 (15458)	29.4 (15458)
Nigeria 2018	4739	75.5 (4739)	-1.42 (4739)	35.3 (4739)	-0.36 (4739)	9.1 (4739)	-1.07 (4739)	23.5 (4739)
Senegal 2010	2440	82.6 (2440)	-1.21 (2440)	28.2 (2440)	-0.53 (2440)	10.3 (2440)	-1.04 (2440)	19.3 (2440)
Senegal 2012	4682	84.1 (4682)	-0.95 (4682)	18.7 (4682)	-0.65 (4682)	10.2 (4682)	-0.96 (4682)	16.8 (4682)
Senegal 2014	4861	84.2 (4861)	-1.01 (4861)	20.1 (4861)	-0.47 (4861)	6.7 (4861)	-0.88 (4861)	13.9 (4861)
Senegal 2015	4790	84.0 (4790)	-1.07 (4790)	20.8 (4790)	-0.56 (4790)	7.8 (4790)	-0.99 (4790)	16.4 (4790)
Senegal 2016	4504	83.4 (4504)	-0.95 (4504)	17.1 (4504)	-0.54 (4504)	7.1 (4504)	-0.91 (4504)	14.3 (4504)
Senegal 2019	3196	76.8 (3196)	-1.03 (3196)	20.9 (3196)	-0.47 (3196)	8.4 (3196)	-0.88 (3196)	15.5 (3196)
Sierra Leone 2008	1235	81.4 (1235)	-1.39 (1235)	35.9 (1235)	-0.22 (1235)	11.4 (1235)	-0.85 (1235)	21.0 (1235)
Sierra Leone 2013	2912	83.0 (2912)	-1.51 (2912)	37.6 (2912)	0.10 (2912)	8.8 (2912)	-0.79 (2912)	15.1 (2912)
Sierra Leone 2019	1822	75.3 (1822)	-1.27 (1822)	30.1 (1822)	-0.06 (1822)	6.8 (1822)	-0.76 (1822)	15.4 (1822)
Togo 2013	881	84.6 (881)	-1.14 (881)	25.3 (881)	-0.22 (881)	5.9 (881)	-0.79 (881)	13.4 (881)
Zambia 2007	2263	82.0 (2263)	-1.82 (2263)	44.9 (2263)	0.33 (2263)	4.6 (2263)	-0.76 (2263)	13.1 (2263)
Zambia 2013	5479	83.5 (5479)	-1.70 (5479)	41.0 (5479)	0.03 (5479)	6.1 (5479)	-0.93 (5479)	15.0 (5479)

DHS: Demographic and Health Survey; DRC: Democratic Republic of the Congo; HAZ: height-for-age z-score; WAZ: weight-for-age z-score; WHZ: weight-for-height z-score

**Table 2:** Anthropometric characteristics of ethnic groups included in the analysis (n = 45).

Ethnic group	N	Height in cm (median, N)	HAZ score (median, N)	Stunted (%, N)	WHZ score (median, N)	Wasted (%, N)	WAZ score (median, N)	Underweight (%, N)
Adja	3054	81.4 (3054)	-1.27 (3054)	31.5 (3054)	-0.21 (3054)	8.9 (3054)	-0.87 (3054)	16.1 (3054)
Akan	1870	85.3 (1870)	-0.99 (1870)	22.6 (1870)	-0.25 (1870)	5.6 (1870)	-0.73 (1870)	11.1 (1870)
Bambara	3467	81.2 (3467)	-1.41 (3467)	36.3 (3467)	-0.51 (3467)	16.0 (3467)	-1.16 (3467)	25.1 (3467)
Bamileke	1152	82.5 (1152)	-0.90 (1152)	21.5 (1152)	0.80 (1152)	1.1 (1152)	0.12 (1152)	2.9 (1152)
Bariba	1307	77.2 (1307)	-1.57 (1307)	36.8 (1307)	-0.17 (1307)	9.1 (1307)	-0.91 (1307)	18.2 (1307)
Bas-Kasai and Kwilu-Kwngo	1578	82.7 (1578)	-1.70 (1578)	41.6 (1578)	-0.39 (1578)	7.8 (1578)	-1.20 (1578)	24.8 (1578)
Basele	2055	81.5 (2055)	-1.98 (2055)	49.7 (2055)	0.05 (2055)	8.3 (2055)	-1.05 (2055)	23.1 (2055)
Bemba	3747	82.4 (3747)	-1.79 (3747)	43.7 (3747)	0.07 (3747)	6.3 (3747)	-0.95 (3747)	16.7 (3747)
Betamaribe	1117	76.8 (1117)	-1.68 (1117)	42.4 (1117)	-0.37 (1117)	12.5 (1117)	-1.15 (1117)	23.2 (1117)
Chewa	3287	79.9 (3287)	-1.78 (3287)	43.7 (3287)	0.23 (3287)	4.6 (3287)	-0.78 (3287)	12.0 (3287)
Cisena	1046	83.6 (1046)	-1.73 (1046)	40.0 (1046)	-0.05 (1046)	7.4 (1046)	-0.93 (1046)	16.7 (1046)
Diola	1015	82.8 (1015)	-1.00 (1015)	20.2 (1015)	-0.22 (1015)	4.0 (1015)	-0.72 (1015)	9.5 (1015)
Dogon	1009	81.6 (1009)	-1.58 (1009)	41.0 (1009)	-0.42 (1009)	12.4 (1009)	-1.10 (1009)	26.1 (1009)
Emakhuwa	1711	81.0 (1711)	-2.07 (1711)	51.7 (1711)	0.21 (1711)	6.0 (1711)	-1.05 (1711)	18.8 (1711)
Fon	4841	79.0 (4841)	-1.43 (4841)	35.6 (4841)	-0.16 (4841)	11.0 (4841)	-0.91 (4841)	17.6 (4841)
Fula	17558	80.4 (17558)	-1.28 (17558)	32.1 (17558)	-0.62 (17558)	13.9 (17558)	-1.17 (17558)	25.2 (17558)
Hausa	12540	78.5 (12540)	-2.12 (12540)	52.3 (12540)	-0.60 (12540)	21.7 (12540)	-1.65 (12540)	39.8 (12540)
Igbo	5752	83.3 (5752)	-0.64 (5752)	17.8 (5752)	-0.26 (5752)	9.4 (5752)	-0.54 (5752)	10.6 (5752)
Ijaw	1554	83.2 (1554)	-1.06 (1554)	26.4 (1554)	0.00 (1554)	6.8 (1554)	-0.55 (1554)	12.1 (1554)
Kalenjin	3251	84.1 (3251)	-1.53 (3251)	35.2 (3251)	-0.28 (3251)	5.9 (3251)	-1.01 (3251)	18.4 (3251)
Kamba	1765	83.8 (1765)	-1.46 (1765)	32.9 (1765)	0.03 (1765)	3.6 (1765)	-0.79 (1765)	12.9 (1765)
Kasai	2881	81.9 (2881)	-1.81 (2881)	46.3 (2881)	-0.18 (2881)	8.4 (2881)	-1.09 (2881)	24.2 (2881)
Kikuyu	2664	86.7 (2664)	-1.12 (2664)	22.3 (2664)	0.14 (2664)	3.1 (2664)	-0.50 (2664)	7.2 (2664)
Kisii	1126	85.0 (1126)	-1.25 (1126)	26.1 (1126)	0.16 (1126)	3.1 (1126)	-0.57 (1126)	10.1 (1126)
Lomwe	1151	79.6 (1151)	-1.68 (1151)	40.0 (1151)	0.21 (1151)	3.4 (1151)	-0.72 (1151)	11.1 (1151)
Luhya	2862	84.6 (2862)	-1.18 (2862)	27.0 (2862)	0.22 (2862)	2.5 (2862)	-0.54 (2862)	8.3 (2862)
Luo	2556	84.4 (2556)	-1.07 (2556)	25.3 (2556)	0.19 (2556)	2.9 (2556)	-0.43 (2556)	8.0 (2556)
Mandinka	5238	81.3 (5238)	-1.15 (5238)	26.9 (5238)	-0.39 (5238)	9.1 (5238)	-0.89 (5238)	17.2 (5238)
Mende	3025	79.7 (3025)	-1.51 (3025)	37.3 (3025)	0.03 (3025)	8.9 (3025)	-0.83 (3025)	16.8 (3025)
Mijikenda	1540	82.9 (1540)	-1.57 (1540)	36.4 (1540)	-0.17 (1540)	6.0 (1540)	-0.95 (1540)	16.7 (1540)
Mole-Dagbani	1222	83.1 (1222)	-1.13 (1222)	24.0 (1222)	-0.46 (1222)	9.4 (1222)	-0.96 (1222)	15.7 (1222)
Mossi	2981	83.1 (2981)	-1.37 (2981)	32.5 (2981)	-0.63 (2981)	16.7 (2981)	-1.19 (2981)	24.2 (2981)
Ngoni	1359	81.9 (1359)	-1.72 (1359)	41.6 (1359)	0.28 (1359)	5.7 (1359)	-0.72 (1359)	12.4 (1359)
Sara	1803	83.0 (1803)	-1.12 (1803)	28.6 (1803)	-0.12 (1803)	8.8 (1803)	-0.74 (1803)	16.5 (1803)
Senoufo	1846	81.0 (1846)	-1.55 (1846)	38.9 (1846)	-0.45 (1846)	12.7 (1846)	-1.22 (1846)	25.6 (1846)
Serere	3228	83.8 (3228)	-0.98 (3228)	18.8 (3228)	-0.49 (3228)	6.4 (3228)	-0.88 (3228)	13.0 (3228)
Soninke/Serahuleh	1959	82.1 (1959)	-1.16 (1959)	28.8 (1959)	-0.58 (1959)	13.4 (1959)	-1.07 (1959)	21.6 (1959)
Temne	2655	79.3 (2655)	-1.29 (2655)	32.5 (2655)	-0.05 (2655)	8.6 (2655)	-0.78 (2655)	16.1 (2655)
Tiv	1176	81.5 (1176)	-1.31 (1176)	31.7 (1176)	0.09 (1176)	7.8 (1176)	-0.63 (1176)	12.8 (1176)
Tonga	2134	82.5 (2134)	-1.60 (2134)	37.8 (2134)	0.06 (2134)	3.9 (2134)	-0.85 (2134)	12.4 (2134)
Tumbuka	1396	81.2 (1396)	-1.69 (1396)	41.3 (1396)	0.32 (1396)	3.6 (1396)	-0.69 (1396)	11.7 (1396)
Ubangi and Itimbiri	1064	81.6 (1064)	-1.60 (1064)	40.4 (1064)	-0.06 (1064)	7.4 (1064)	-0.93 (1064)	18.1 (1064)
Wollof	9089	82.7 (9089)	-0.94 (9089)	18.4 (9089)	-0.53 (9089)	8.0 (9089)	-0.89 (9089)	14.0 (9089)
Xichangana	1462	83.2 (1462)	-1.22 (1462)	25.3 (1462)	0.47 (1462)	2.3 (1462)	-0.34 (1462)	7.3 (1462)
Yoruba	7219	81.9 (7219)	-1.14 (7219)	27.9 (7219)	-0.24 (7219)	9.6 (7219)	-0.80 (7219)	14.6 (7219)

DRC: Democratic Republic of the Congo; HAZ: height-for-age z-score; WAZ: weight-for-age z-score; WHZ: weight-for-height z-score

### Association between wasting and ethnicity

**Fig 5:**
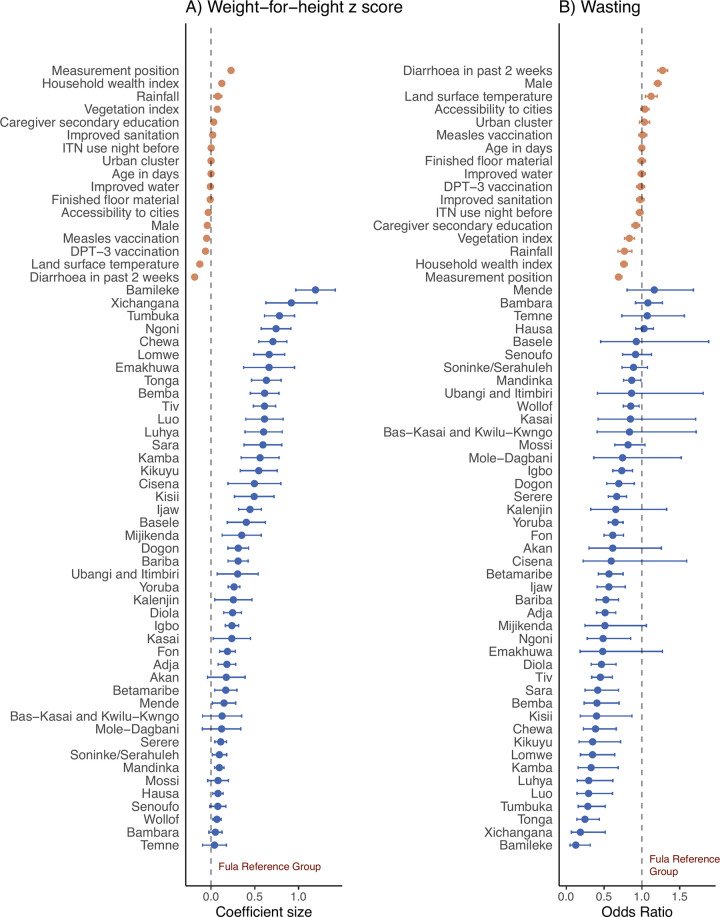
Association between ethnicity and (A) weight-for-height *z* (WHZ) score and (B) wasting among children aged <5 years in 18 countries in sub-Saharan Africa surveyed between 2006 to 2019. Effect estimates and 95% confidence intervals (CI) are from a generalised linear hierarchical mixed effects model controlling for all variables shown. Wasting is defined as a WAZ score more than two standard deviations lower than the World Health Organisation reference median [36].

A total of 13,741 (9.9%) of 138,312 children were wasted and 58% of children had a weight-for-height lower than the WHO median. Wasting prevalence ranged from 1.1% (13 of 1,152) in Bamileke children to 21.7% (2,720 of 12,540) in Hausa children (Tables 1 and 2). WHZ scores differed by at least 0.5 SD in 36% of significant pairwise comparisons between ethnic groups. The largest difference between any two ethnic groups was 1.19 SD (S1 Fig). Compared to Fula children, the adjusted difference in WHZ associated with ethnicity ranged from 0.07 SD higher (95% CI 0.02–0.12, p = 0.0056) in Wollof children to 1.19 SD higher (95% CI 0.97–1.42, p<0.0001) in Bamileke children. Compared to Fula children, the difference in odds of wasting associated with ethnicity ranged from 87% lower odds in Bamileke children (aOR 0.13, 95% CI 0.05–0.32, p<0.0001) to 13% lower odds in Mandinka children (aOR 0.87, 95% CI 0.76–0.99, p = 0.034) (Fig 5 and S3 Table). As a comparison, the association between wasting and other covariables ranged from 31% lower odds of wasting in children with height measured in a standing, compared to lying, position (aOR 0.69, 95% CI 0.65–0.73, p<0.0001) to 27% higher odds of wasting in children who had a reported episode of diarrhoea in the past two weeks compared to no episode (aOR 1.27, 95% CI 1.21–1.34, p<0.0001).

### Association between underweight and ethnicity

**Fig 6:**
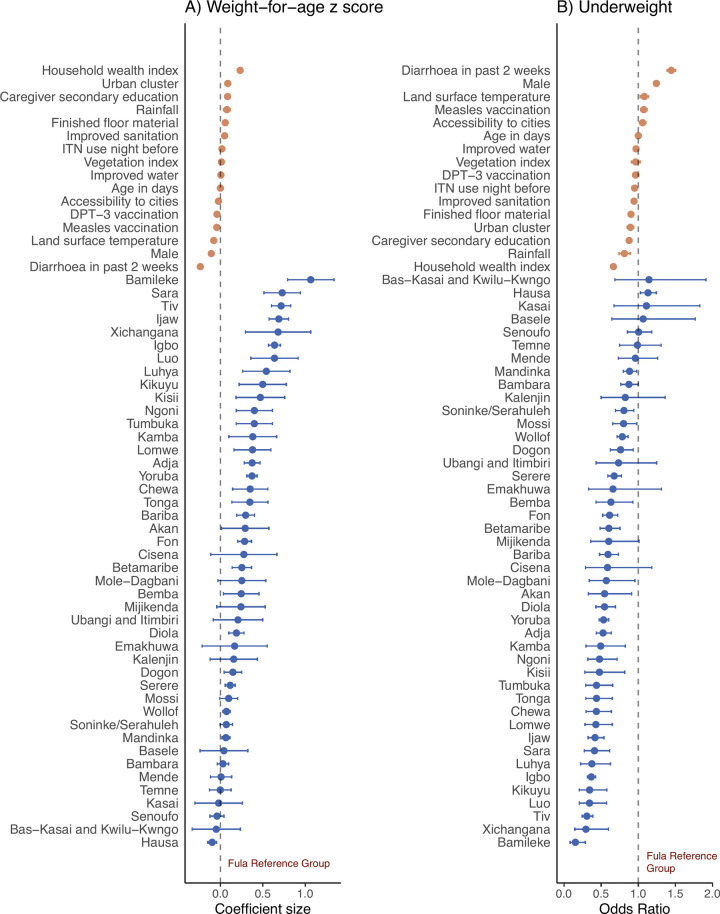
Association between ethnicity and (a) weight-for-age *z* (WAZ) score and (b) underweight among children aged <5 years in 18 countries in sub-Saharan Africa surveyed between 2006 to 2019. Effect estimates and 95% confidence intervals (CI) are from a generalised linear hierarchical mixed effects model controlling for all variables shown. Underweight is defined as a WAZ score more than two standard deviations lower than the World Health Organisation reference median [36].

A total of 26,297 (19.0%) of 138,312 children were underweight and 79% of children had a weight-for-height lower than the WHO median. Underweight prevalence ranged from 2.9% (33 of 1,152) in Bamileke children to 39.8% (4,994 of 12,540) in Hausa children (Tables 1 and 2). WAZ scores differed by at least 0.5 SD in 20% of significant pairwise comparisons between ethnic groups. The largest difference between any two ethnic groups was 1.16 SD (S1 Fig). Compared to Fula children, the adjusted difference in WAZ score associated with ethnicity ranged from 1.06 SD higher (95% CI 0.79–1.34, p<0.0001) in Bamileke children to 0.10 SD lower (95% CI -0.15, -0.04, p<0.00027) in Hausa children. Compared to Fula children, the adjusted difference in odds of underweight associated with ethnicity ranged from 85% lower odds in Bamileke children (aOR 0.15, 95% CI 0.08–0.29, p<0.0001) to 13% higher odds in Hausa children (aOR 1.13, 95% CI 1.03–1.24, p = 0.010) (Fig 6 and S3 Table). As a comparison, the association between underweight and other covariables ranged from 34% lower odds of underweight in wealthier households compared to less wealthy households (aOR 0.66, 95% CI 0.64–0.69, p<0.0001) to 44% higher odds of underweight in children who had a reported episode of diarrhoea in the past two weeks, compared to no episode (aOR 1.44, 95% CI 1.39–1.50, p<0.0001).

## Discussion

We investigated whether there are significant differences in anthropometric deficits in children belonging to different ethnic groups across SSA using nationally representative survey data. By analysing data for 138,312 children aged <5 years across 18 countries, we found that ethnicity is closely associated with child anthropometric deficits, accounting for differences of up to 1.30 SD in HAZ, 1.19 SD in WHZ and 1.16 SD in WAZ after adjusting for other covariables. These differences are striking, considering that an anthropometric deficit is represented by a z-score greater than 2 SD below the WHO 2006 reference median and a difference of 0.5 SD is considered clinically significant [11]. No other covariables had associations of comparable magnitude with the malnutrition scores. Our findings suggest that ethnicity is an important factor linked to anthropometric deficits in children.

Few multi-country studies have measured differences in child growth outcomes across ethnic groups. An analysis of national survey data from Latin America found that the prevalence of stunting was on average 34% higher among indigenous children aged <5 years compared to a reference group of European or mixed ancestry children, after adjusting for wealth and place of residence (prevalence ratio 1.34, 95% CI 1.28–1.39, p = 0.009) [6]. A 2014 systematic review comparing data from the WHO MGRS with data from studies performed in 55 countries or ethnic groups also found variation in height across ethnic groups [7]. Within local studies, ethnic group differences in child growth outcomes have been found in China [21], Malaysia [22], Nepal [23], South Africa [24], the UK [25] and the USA, but not in other studies including in Thailand [26] and South Africa [27]. Our study is consistent with previous studies that suggest that the burden of anthropometric deficits may be unequally distributed among some ethnic groups.

Ethnicity, a characteristic that reflects shared genetic heritage and culture, may be associated with anthropometric deficits through several environmental factors. First, ethnicity may be linked to socioeconomic risk factors for undernutrition including poor diet; inadequate water source, sanitation and hygiene; and repeat episodes of infectious disease. Overall, we found children’s height-for-age, weight-for-age and weight-for-height to be significantly lower on average among African children than the WHO median; 80% of children had HAZ scores less than zero. This finding is consistent with current evidence that body size is generally lower in LMICs than in high-income countries [28]. Despite this, we found that ethnic group disparities persisted after adjusting for socioeconomic factors. Second, ethnicity may be linked to different cultural practices and behaviours that influence growth such as breastfeeding rates, sleep and diurnal rhythm and patterns of lifestyle and diet [29]. For example we found growth faltering to be more common among Fula than Mandinka, Wollof and Serere children, consistent with an earlier observation that stunting is more prevalent among Fula than Mandinka children in The Gambia [30]. The Fula have specific food cultures that may impede maternal and child nutrition; for example, pregnant Fula women do not consume important protein sources such as eggs, catfish or groundnuts [30]. Third, ethnicity may be linked to differential access to and uptake of health care and essential services such as vaccinations. Ethnicity is a known risk factor for other child health outcomes including under five year mortality across SSA [5] and tackling the causes of such inequalities is important to attain SDG 2.

Growth is a function of both environmental and biological potential and it is important to understand how both of these may mediate the association between ethnicity and anthropometric deficits, including the interaction between genes, hormones and the environment [29]. For example, a recent analysis found that the odds of wasting among children were 27% higher in the hottest parts compared to the coolest parts of Africa [16]. Population-level differences in subscapular skinfold (which affects heat loss) are also present [31]. It is possible that growth limitation partly reflects a survival response to extreme temperatures driven by the energetic cost of thermoregulation caused by heat stress [32] and an adaptation towards heat loss, for example through higher area-to-mass ratios [33]. Other biological processes may be important. Sleep, for example, is not only affected by behaviour, but there is an increased occurrence of single-nucleotide polymorphisms in some African populations that reduce the duration and quality of sleep [34]. Research is needed to understand these interconnecting vulnerabilities and their relationship with children’s growth.

The association between ethnicity and anthropometric deficits, if causative, points to the need to understand the implications for monitoring growth. Stunting and wasting, key metrics used to assess nutritional status and evaluate progress towards nutrition goals, are identified using the WHO Child Growth Standards (WHO-CGS), a universal standard based on the WHO MGRS 1997–2003 study conducted in Brazil, Ghana, India, Norway, Oman and the USA [11]. Since identification of anthropometric deficits varies according to the growth standard used, not all countries have adopted the WHO-CGS [35]. Such local references are more difficult to develop and implement than a universal reference, but accurate measurement of stunting and wasting is essential to ensure that anthropometric deficits are not under- or overestimated in individual children and to enable research into the putative causes and consequences of these outcomes.

Our study has several limitations. The causes of growth faltering are complicated and our analysis provides only an initial exploration of the role of ethnicity based on observational (cross-sectional) data that cannot determine causality. While we adjusted for a range of covariables, residual confounding of the relationship between ethnicity and growth outcomes by social and environmental factors is possible, and we did not adjust for nutritional intake. The measures of temperature, vegetation index and rainfall used were based on synoptic means from 2000 to 2016 which are imprecise metrics of climate conditions. Our study did not include sub-group analyses of gender, age and survey time period, nor compared ethnic inequalities within and between countries; these investigations would be valuable in future studies. Overall, we do not aim to present a comprehensive investigation, but a preliminary analysis that quantifies the relationship between ethnicity and anthropometric deficits and stimulates further work in this area.

In conclusion, significant ethnic disparities in stunting, wasting and underweight exist across SSA. Understanding and accounting for these differences is important to support effective progress monitoring and targeting of nutrition interventions.

## Supporting information

S1 Checklist STROBE statement. (PDF)

S1 Text Model performance. (PDF)

S1 Table Household- and child-level characteristics of surveys included in the analysis (n = 37). (PDF)

S2 Table Household- and child-level characteristics of ethnic groups included in the analysis (n = 45). (PDF)

S3 Table Association between ethnicity and growth outcomes in children (n = 138,312) aged <5 years in sub-Saharan Africa. (PDF)

S1 Fig Growth variation relative to ethnic group among 138,312 children aged <5 years in 18 countries in sub-Saharan Africa surveyed between 2006 to 2019. Figures show significant pair-wise comparisons of mean weight-for-height z-scores and weight-for-age z-scores between ethnic groups.
(PDF)

S2 Fig Model validation for height-for-age (HAZ) z-score and stunting in relation to ethnicity. AUC: Area under curve; cor: correlation.
(PDF)

S3 Fig Model validation for weight-for-height (WHZ) z-score and wasting in relation to ethnicity. AUC: Area under curve; cor: correlation.
(PDF)

S4 Fig Model validation for weight-for-age (WAZ) z-score and underweight in relation to ethnicity. AUC: Area under curve; cor: correlation.
(PDF)
