# Supplementary material for: Ethnicity and anthropometric deficits in children: A cross-sectional analysis of national survey data from 18 countries in sub-Saharan Africa
Source: PLOS Glob Public Health. 2024 Dec 31;4(12):e0003067. doi: 10.1371/journal.pgph.0003067 (PMC11687787; doi:10.1371/journal.pgph.0003067)
Supplement: S1 Text — (PDF) [file pgph.0003067.s002.pdf]

S1. Text. Model performance details

Model performance was evaluated by calculating the area under the receiver operator characteristic curve comparing the child-level (not cluster-level) predictions for each child against the actual. For height-for-age z-scores an area under the curve (AUC) of 0.749 (0.737 – 0.743) was found, for weight-for-height z-scores an AUC of 0.791 (0.788 – 0.795) was found and for weight-for-age z-scores an AUC of 0.751 (0.748-0.754) was found. These scores indicate an adequate predictive performance at the child level. Model calibration was assessed using Q-Q plots, and the plots demonstrated a close alignment with a straight line, indicating that the distribution of the model's predictions closely matches the distribution of the observed data. This suggests that the model is well-calibrated with respect to the quantiles and distributional characteristics of the data. Predictive scores (S2-S4 Figures) indicated good performance at both child and ethnicity level. As expected, there was uncertainty in predicting growth failure in any individual child, but when examined at a population level by ethnicity this uncertainty shrunk greatly and showed a strong relationship that generalised out of sample (as measured by cross validation).
