# Supplementary material for: Ethnicity and anthropometric deficits in children: A cross-sectional analysis of national survey data from 18 countries in sub-Saharan Africa
Source: PLOS Glob Public Health. 2024 Dec 31;4(12):e0003067. doi: 10.1371/journal.pgph.0003067 (PMC11687787; doi:10.1371/journal.pgph.0003067)
Supplement: S1 Table — (PDF) [file pgph.0003067.s003.pdf]

Table S1. Household- and child-level characteristics of surveys included in the analysis (n=37)

		Household-level characteristics					Child-level characteristics							
**DHS Survey**	**N**	**Household head attended secondary education (%)**	**Urban residence (%)**	**Finished floor material (%)**	**Improved drinking water source (%)**	**Improved sanitation (%)**	**N**	**Mean age in years (%)**	**Male (%)**	**Height measured standing (not lying) (%)**	**Received DPT-3 vaccine (%)**	**Received measles-1 vaccine (%)**	**Reported ITN use previous night (%)**	**Reported diarrhoea in past two weeks (%)**
Benin 2012	4023	23.9	36.7	60.0	80.1	26.8	5589	2.5	49.8	72.1	64.4	66.6	77.2	7.6
Benin 2017	4787	24.3	39.7	65.0	71.0	29.2	5975	1.4	49.4	30.3	62.5	47.3	72.2	14.2
Burkina Faso 2010	2466	7.1	27.6	48.3	82.6	31.7	3873	2.3	51.6	61.7	80.9	72.3	53.4	14.8
Cameroon 2011	596	55.0	73.2	73.2	82.2	76.7	926	2.3	50.1	49.9	80.2	75.3	32.9	13.9
Cameroon 2018	196	52.6	62.2	69.9	78.6	77.6	245	1.5	45.7	35.9	75.9	63.3	0.0	12.2
Chad 2014	1206	33.9	17.5	5.9	42.4	9.5	1886	2.3	49.2	52.4	35.3	56.7	41.3	26.8
DRC 2007	1385	54.4	40.1	17.8	43.7	38.6	2099	2.3	48.3	53.9	45.1	56.4	11.8	16.7
DRC 2013	3332	57.7	31.2	11.1	39.3	34.6	5479	2.4	50.0	55.6	50.9	59.7	57.9	17.6
Cote d'Ivoire 2012	405	12.3	44.4	71.1	86.9	49.6	644	2.2	48.6	38.5	51.7	56.4	39.6	18.2
Ghana 2008	1038	54.3	38.9	81.9	82.2	61.8	1385	2.4	50.8	65.1	80.4	77.8	44.3	21.6
Ghana 2014	1255	57.1	44.7	92.5	75.1	66.4	1707	2.3	50.3	55.5	80.8	74.1	48.8	12.6
Guinea 2012	1369	14.5	28.3	51.3	72.9	37.6	2150	2.3	52.5	58.6	38.4	51.6	33.2	18.0
Guinea 2018	1179	17.8	27.2	58.7	74.7	46.7	1416	1.4	50.5	30.7	27.8	24.6	21.8	15.5
Kenya 2008	2577	33.6	26.4	38.7	62.1	44.6	3696	2.4	50.2	59.6	76.3	70.8	55.4	18.1
Kenya 2014	8923	36.6	35.7	40.4	64.5	46.8	12068	2.4	50.6	57.3	85.0	75.1	61.4	15.9
Liberia 2013	76	28.9	57.9	40.8	89.5	55.3	118	2.4	50.8	56.8	75.4	63.6	50.8	15.3
Malawi 2010	2267	23.0	9.9	18.2	78.1	14.4	3065	2.4	50.6	67.9	87.9	81.5	49.5	17.1
Malawi 2015	2106	29.4	15.7	21.9	83.5	80.6	2267	1.5	49.9	35.2	84.1	70.7	51.3	28.4
Mali 2006	3554	11.5	32.9	29.3	58.8	22.1	5339	2.2	50.8	60.6	63.9	60.9	39.2	13.1
Mali 2012	2327	12.2	25.0	27.7	65.0	41.5	3612	2.5	51.0	61.7	58.5	63.6	73.8	8.7
Mali 2018	500	19.2	36.4	52.4	71.6	57.8	570	1.4	46.7	33.0	59.6	51.2	0.0	17.0
Mozambique 2011	2864	13.9	37.3	30.5	56.7	24.0	4219	2.3	49.6	57.9	75.6	74.5	36.4	10.3
Nigeria 2008	7145	37.3	32.9	55.8	54.5	52.4	10722	2.3	49.2	61.2	33.7	37.8	7.2	10.0
Nigeria 2013	9848	39.2	41.2	58.8	58.5	52.7	15458	2.4	50.1	53.7	35.7	36.9	19.8	10.4
Nigeria 2018	3928	50.2	45.5	71.1	61.9	53.1	4739	1.5	51.5	32.7	47.0	42.8	0.0	15.2
Senegal 2010	1314	8.3	31.8	54.3	65.9	43.4	2440	2.2	52.9	61.1	75.8	71.4	46.9	20.9
Senegal 2012	2247	9.6	32.4	64.0	68.3	48.9	4682	2.3	50.7	60.2	81.0	69.2	53.1	15.0

Table S1. Household- and child-level characteristics of surveys included in the analysis (n=37)

		Household-level characteristics					Child-level characteristics							
**DHS Survey**	**N**	**Household head attended secondary education (%)**	**Urban residence (%)**	**Finished floor material (%)**	**Improved drinking water source (%)**	**Improved sanitation (%)**	**N**	**Mean age in years (%)**	**Male (%)**	**Height measured standing (not lying) (%)**	**Received DPT-3 vaccine (%)**	**Received measles-1 vaccine (%)**	**Reported ITN use previous night (%)**	**Reported diarrhoea in past two weeks (%)**
Senegal 2014	2320	8.6	32.5	60.1	70.1	45.9	4861	2.3	50.2	62.1	81.5	69.2	52.5	20.7
Senegal 2015	2357	7.8	30.8	67.7	65.1	46.0	4790	2.3	50.6	57.8	80.7	66.6	58.0	21.7
Senegal 2016	2355	9.3	31.8	69.2	74.6	50.6	4504	2.3	52.6	54.1	82.2	67.6	71.3	17.9
Senegal 2019	2060	11.6	29.8	74.3	71.9	63.1	3196	1.5	49.8	33.2	78.6	62.2	70.2	16.6
Sierra Leone 2008	886	23.1	32.4	32.3	52.0	40.4	1235	2.3	48.6	58.0	55.0	55.6	35.3	11.7
Sierra Leone 2013	2052	22.2	31.6	37.8	58.2	49.1	2912	2.4	47.5	61.9	72.8	73.2	52.2	11.1
Sierra Leone 2019	1573	30.8	30.7	47.1	60.5	48.4	1822	1.4	50.2	31.0	68.2	53.4	0.0	8.4
Togo 2013	640	43.1	49.5	88.4	63.9	48.3	881	2.4	49.5	57.4	72.5	63.2	42.0	15.1
Zambia 2007	1544	39.8	37.2	38.7	41.5	36.9	2263	2.4	49.1	54.3	34.1	72.8	34.3	14.8
Zambia 2013	3888	47.0	41.6	41.6	63.1	44.1	5479	2.5	50.1	58.0	81.6	75.1	42.8	15.5

DHS: Demographic and Health Survey; DPT: diptheria-pertussis-tetanus; DRC: Democratic Republic of the Congo; ITN: insecticide-treated net
