# Supplementary material for: Ethnicity and anthropometric deficits in children: A cross-sectional analysis of national survey data from 18 countries in sub-Saharan Africa
Source: PLOS Glob Public Health. 2024 Dec 31;4(12):e0003067. doi: 10.1371/journal.pgph.0003067 (PMC11687787; doi:10.1371/journal.pgph.0003067)
Supplement: S2 Table — (PDF) [file pgph.0003067.s004.pdf]

		**Household-level characteristics**						**Child-level characteristics**							
**Ethnic group**	**Countries represented**	**N**	**Household head attended secondary education (%)**	**Urban residence (%)**	**Finished floor material (%)**	**Improved drinking water source (%)**	**Improved sanitation (%)**	**N**	**Mean age in years (%)**	**Male (%)**	**Height measured standing (not lying) [1] (%)**	**Received DPT-3 vaccine (%)**	**Received measles-1 vaccine (%)**	**Reported ITN use previous night (%)**	**Reported diarrhoea in past two weeks (%)**
Adja	Benin, Togo	2274	33.3	38.0	65.2	72.9	32.8	3054	2.2	50.1	53.2	64.2	58.9	67.9	13.4
Akan	Ghana	1400	73.5	45.7	93.6	73.5	80.0	1870	2.4	49.8	61.9	81.0	77.2	47.3	15.0
Bambara	Mali	2389	12.3	30.8	29.6	62.9	31.7	3467	2.3	50.1	58.1	65.6	63.5	47.8	10.6
Bamileke	Cameroon	775	55.1	70.6	72.5	81.5	77.4	1152	2.2	49.1	47.2	79.9	73.4	26.5	13.3
Bariba	Benin	970	16.3	43.1	66.0	57.0	15.9	1307	1.8	48.8	43.0	65.7	56.2	69.0	14.4
Bas-Kasai and Kwilu-Kwngo	DRC	1009	67.0	37.2	17.3	33.8	30.6	1578	2.4	49.4	55.7	60.8	67.9	61.2	14.3
Basele	DRC	1294	46.0	30.1	9.6	56.3	42.5	2055	2.4	48.8	56.6	53.3	62.7	33.4	16.4
Bemba	Zambia	2640	49.1	48.0	39.8	51.4	43.8	3747	2.4	49.7	57.4	69.0	72.8	42.0	15.2
Betamaribe	Benin	799	14.0	30.4	41.9	66.1	5.9	1117	1.9	49.4	52.4	70.9	59.0	69.8	16.7
Chewa	Malawi, Zambia	2593	25.6	16.0	22.1	74.0	43.1	3287	2.2	49.6	55.3	82.0	76.0	48.7	20.2
Cisena	Mozambique	627	15.8	28.5	12.3	49.4	17.4	1046	2.4	47.6	56.5	73.1	72.0	36.5	13.2
Diola	Burkina Faso, Cote d'Ivoire, Senegal	694	22.6	38.2	63.4	48.0	50.6	1015	2.2	50.4	56.5	84.8	74.2	65.3	19.5
Dogon	Mali	668	8.8	21.6	17.8	53.7	19.3	1009	2.4	48.8	61.2	51.4	57.0	56.2	9.4
Emakhuwa	Mozambique	1181	11.3	27.3	7.6	39.1	15.5	1711	2.3	51.2	60.9	70.0	72.6	52.3	8.2
Fon	Benin	3826	27.9	41.2	70.8	80.5	38.3	4841	2.0	49.5	55.2	65.6	60.5	78.6	7.9
Fula	Benin, Burkina Faso, Chad, Guinea, Mali, Nigeria, Senegal, Sierra Leone	11172	10.0	25.3	45.9	58.0	36.1	17558	2.1	50.5	53.2	52.7	48.4	42.4	17.8
Hausa	Nigeria	8012	24.3	25.6	42.9	54.9	56.7	12540	2.2	49.2	53.3	14.3	21.6	11.5	13.5
Igbo	Nigeria	3951	51.5	59.8	81.0	68.2	61.4	5752	2.2	51.0	54.5	71.6	61.9	16.5	7.4
Ijaw	Nigeria	1066	65.8	22.9	72.7	32.0	14.8	1554	2.2	50.8	48.5	40.9	45.9	17.0	2.8
Kalenjin	Kenya	2254	32.3	19.0	25.1	46.8	40.2	3251	2.4	51.1	55.5	84.7	72.6	50.8	12.5
Kamba	Kenya	1336	31.4	37.4	49.3	53.0	50.1	1765	2.4	50.7	56.5	86.7	77.0	58.9	15.1
Kasai	DRC	1763	59.6	38.2	15.4	40.0	31.5	2881	2.3	49.3	53.5	45.8	52.1	39.7	20.6
Kikuyu	Kenya	2232	47.0	44.2	57.0	76.5	58.6	2664	2.5	51.5	58.4	88.2	80.8	48.5	10.4
Kisii	Kenya	870	49.1	31.8	36.1	74.6	41.7	1126	2.5	51.8	62.2	86.4	77.2	69.4	11.7
Lomwe	Malawi	972	24.1	14.7	19.7	86.9	47.5	1151	2.0	50.5	52.2	87.6	77.9	48.7	23.6
Luhya	Kenya	2039	35.8	33.1	33.3	76.3	38.9	2862	2.4	50.5	59.1	81.6	72.7	68.0	20.5
Luo	Kenya	1782	34.1	39.8	45.7	59.1	49.2	2556	2.4	49.8	58.1	74.7	69.0	67.9	22.5
Mandinka	Cote d'Ivoire, Guinea, Liberia, Mali, Senegal, Sierra Leone	3108	17.5	36.0	54.3	68.5	42.7	5238	2.1	51.4	53.5	61.7	57.5	49.1	18.2
Mende	Liberia, Sierra Leone	2315	25.4	25.8	33.8	59.9	41.1	3025	2.1	48.9	52.6	72.4	67.3	37.3	8.2

		Household-level characteristics						Child-level characteristics							
**Ethnic group**	**Countries represented**	**N**	**Household head attended secondary education (%)**	**Urban residence (%)**	**Finished floor material (%)**	**Improved drinking water source (%)**	**Improved sanitation (%)**	**N**	**Mean age in years (%)**	**Male (%)**	**Height measured standing (not lying) [1] (%)**	**Received DPT-3 vaccine (%)**	**Received measles-1 vaccine (%)**	**Reported ITN use previous night (%)**	**Reported diarrhoea in past two weeks (%)**
Mijikenda	Kenya	987	17.2	29.8	30.1	63.3	41.4	1540	2.4	48.1	57.3	79.8	71.2	66.0	22.4
Mole-Dagbani	Ghana	893	28.1	36.4	78.4	85.8	39.6	1222	2.3	51.6	56.5	80.0	73.6	46.0	19.1
Mossi	Burkina Faso	1889	7.5	28.8	53.1	83.2	34.3	2981	2.3	52.1	62.8	83.5	73.9	54.8	14.3
Ngoni	Malawi, Zambia	1087	31.5	27.5	32.8	79.2	49.9	1359	2.2	51.4	55.2	81.3	79.4	48.1	17.2
Sara	Cameroon, Chad	1156	35.2	17.6	6.5	41.9	9.5	1803	2.3	49.3	52.4	36.4	57.7	40.8	27.0
Senoufo	Burkina Faso, Cote d'Ivoire, Mali	1144	11.6	29.2	41.3	70.0	28.8	1846	2.2	52.2	52.7	64.5	63.9	49.1	16.2
Serere	Senegal	1674	10.0	30.2	66.1	69.9	53.0	3228	2.3	51.5	59.0	83.4	70.6	59.4	17.1
Soninke/ Serahuleh	Mali, Senegal	1148	10.6	37.2	44.9	66.5	42.3	1959	2.3	49.1	61.7	65.8	64.3	52.2	13.7
Temne	Sierra Leone	1947	26.0	34.6	43.0	54.7	51.5	2655	2.0	48.0	49.8	62.8	58.7	28.5	12.7
Tiv	Nigeria	768	55.1	11.5	43.5	49.3	22.7	1176	2.2	50.3	52.7	26.7	32.7	9.3	8.8
Tonga	Malawi, Zambia	1468	42.0	26.0	39.8	62.7	39.2	2134	2.4	49.1	55.1	65.7	75.1	38.6	16.9
Tumbuka	Malawi, Zambia	1045	40.9	23.3	32.1	69.0	43.7	1396	2.2	51.6	56.7	75.1	75.7	42.3	17.8
Ubangi and Itimbiri	DRC	651	54.2	23.7	7.1	21.5	41.8	1064	2.4	51.8	56.1	34.0	55.8	58.6	14.7
Wollof	Senegal	4161	7.7	33.0	75.8	83.4	65.0	9089	2.2	51.2	54.6	83.0	70.0	56.7	17.8
Xichangana	Mozambique	1056	15.6	53.7	66.9	80.7	37.5	1462	2.3	49.2	55.4	84.0	78.5	17.9	10.7
Yoruba	Benin, Nigeria	5503	60.2	63.2	86.4	76.3	55.8	7219	2.2	50.7	51.8	67.2	61.1	22.8	7.7

DPT: diptheria-pertussis-tetanus; DRC: Democratic Republic of the Congo; ITN: insecticide-treated net

[1] If the child is less than two years old, length (recumbent position) is measured. If the child is two years of age or older, height (standing position) is measured. If accurate age is not possible to obtain, length is measured if the child is less than 85 cm. Height is measured if the child is equal to or greater than 85 cm.
