# Supplementary material for: Ethnicity and anthropometric deficits in children: A cross-sectional analysis of national survey data from 18 countries in sub-Saharan Africa
Source: PLOS Glob Public Health. 2024 Dec 31;4(12):e0003067. doi: 10.1371/journal.pgph.0003067 (PMC11687787; doi:10.1371/journal.pgph.0003067)
Supplement: S3 Table — (PDF) [file pgph.0003067.s005.pdf]

Table S3. Association between ethnicity and growth outcomes in children (n=138,312) aged <5 years in sub-Saharan Africa

	**Stunting**		**Wasting**		**Underweight**		**HAZ score**		**WHZ score**		**WAZ score**	
	OR (95% CI)	p	OR (95% CI)	p	OR (95% CI)	p	Coefficient (95% CI)	p	Coefficient (95% CI)	p	Coefficient (95% CI)	p
Ethnic group												
Adja	0.55 (0.46, 0.64)	<0.0001	0.51 (0.40, 0.66)	<0.0001	0.53 (0.43, 0.64)	<0.0001	0.49 (0.36, 0.61)	<0.0001	0.18 (0.08, 0.28)	0.00044	0.37 (0.28, 0.47)	<0.0001
Akan	0.64 (0.34, 1.20)	0.16	0.62 (0.30, 1.26)	0.18	0.55 (0.33, 0.91)	0.020	0.32 (-0.07, 0.71)	0.10	0.17 (-0.04, 0.39)	0.11	0.29 (0.01, 0.57)	0.041
Bambara	1.00 (0.88, 1.13)	0.95	1.08 (0.92, 1.27)	0.36	0.87 (0.76, 1.00)	0.048	-0.02 (-0.11, 0.07)	0.68	0.05 (-0.02, 0.12)	0.19	0.03 (-0.04, 0.10)	0.38
Bamileke	0.59 (0.32, 1.09)	0.092	0.13 (0.05, 0.32)	<0.0001	0.15 (0.08, 0.29)	<0.0001	0.41 (0.03, 0.78)	0.036	1.19 (0.97, 1.42)	<0.0001	1.06 (0.79, 1.34)	<0.0001
Bariba	0.73 (0.61, 0.87)	0.00053	0.52 (0.40, 0.69)	<0.0001	0.59 (0.48, 0.73)	<0.0001	0.19 (0.05, 0.33)	0.0085	0.31 (0.19, 0.42)	<0.0001	0.30 (0.19, 0.40)	<0.0001
Bas-Kasai and Kwilu-Kwngo	1.32 (0.70, 2.48)	0.39	0.84 (0.41, 1.72)	0.63	1.14 (0.69, 1.91)	0.61	-0.17 (-0.56, 0.23)	0.41	0.13 (-0.10, 0.35)	0.27	-0.05 (-0.33, 0.24)	0.73
Basele	1.72 (0.92, 3.21)	0.092	0.93 (0.46, 1.88)	0.83	1.07 (0.64, 1.77)	0.81	-0.35 (-0.74, 0.04)	0.08	0.40 (0.19, 0.62)	0.00028	0.04 (-0.24, 0.32)	0.78
Bemba	1.42 (0.89, 2.27)	0.14	0.41 (0.24, 0.70)	0.0011	0.63 (0.43, 0.93)	0.019	-0.30 (-0.59, -0.00)	0.047	0.61 (0.45, 0.78)	<0.0001	0.25 (0.04, 0.45)	0.022
Betamaribe	0.71 (0.59, 0.86)	0.00055	0.57 (0.43, 0.75)	<0.0001	0.60 (0.48, 0.75)	<0.0001	0.26 (0.11, 0.41)	0.00069	0.17 (0.04, 0.30)	0.0094	0.25 (0.14, 0.37)	<0.0001
Chewa	1.41 (0.89, 2.24)	0.14	0.39 (0.23, 0.66)	0.00052	0.44 (0.30, 0.64)	<0.0001	-0.23 (-0.52, 0.05)	0.11	0.71 (0.55, 0.87)	<0.0001	0.35 (0.14, 0.56)	0.00091
Cisena	1.07 (0.45, 2.56)	0.87	0.59 (0.22, 1.59)	0.30	0.59 (0.29, 1.18)	0.14	-0.06 (-0.60, 0.49)	0.84	0.50 (0.19, 0.80)	0.0013	0.28 (-0.11, 0.67)	0.17
Diola	0.84 (0.70, 1.01)	0.071	0.47 (0.33, 0.66)	<0.0001	0.55 (0.43, 0.69)	<0.0001	0.04 (-0.08, 0.16)	0.50	0.25 (0.14, 0.35)	<0.0001	0.19 (0.10, 0.28)	<0.0001
Dogon	1.15 (0.96, 1.38)	0.13	0.70 (0.54, 0.90)	0.0059	0.76 (0.62, 0.93)	0.0080	-0.11 (-0.25, 0.03)	0.11	0.31 (0.19, 0.43)	<0.0001	0.15 (0.04, 0.25)	0.0058
Emakhuwa	1.72 (0.73, 4.07)	0.22	0.48 (0.18, 1.27)	0.14	0.66 (0.33, 1.31)	0.23	-0.46 (-0.99, 0.07)	0.092	0.66 (0.37, 0.95)	<0.0001	0.17 (-0.22, 0.55)	0.39
Fon	0.66 (0.57, 0.76)	<0.0001	0.62 (0.50, 0.76)	<0.0001	0.61 (0.52, 0.72)	<0.0001	0.30 (0.19, 0.41)	<0.0001	0.19 (0.10, 0.28)	<0.0001	0.28 (0.20, 0.37)	<0.0001
Fula	Reference	-	Reference	-	Reference	-	Reference	-	Reference	-	Reference	-
Hausa	1.32 (1.21, 1.44)	<0.0001	1.03 (0.92, 1.15)	0.61	1.13 (1.03, 1.24)	0.010	-0.29 (-0.36, -0.22)	<0.0001	0.08 (0.02, 0.14)	0.0072	-0.10 (-0.15, -0.04)	0.00027
Igbo	0.31 (0.27, 0.35)	<0.0001	0.74 (0.62, 0.88)	0.00059	0.37 (0.32, 0.43)	<0.0001	0.84 (0.75, 0.93)	<0.0001	0.24 (0.16, 0.32)	<0.0001	0.64 (0.57, 0.71)	<0.0001
Ijaw	0.41 (0.33, 0.50)	<0.0001	0.56 (0.41, 0.78)	0.00054	0.42 (0.32, 0.54)	<0.0001	0.69 (0.54, 0.84)	<0.0001	0.45 (0.32, 0.57)	<0.0001	0.69 (0.57, 0.80)	<0.0001
Kalenjin	0.98 (0.52, 1.82)	0.94	0.66 (0.32, 1.33)	0.24	0.82 (0.50, 1.36)	0.45	-0.01 (-0.40, 0.38)	0.96	0.26 (0.04, 0.47)	0.019	0.16 (-0.12, 0.44)	0.27
Kamba	0.97 (0.52, 1.82)	0.93	0.33 (0.16, 0.69)	0.0030	0.49 (0.30, 0.83)	0.0071	0.01 (-0.39, 0.40)	0.98	0.56 (0.34, 0.78)	<0.0001	0.38 (0.10, 0.66)	0.0079
Kasai	1.64 (0.88, 3.05)	0.12	0.85 (0.42, 1.71)	0.65	1.11 (0.67, 1.83)	0.68	-0.26 (-0.65, 0.12)	0.18	0.24 (0.02, 0.45)	0.030	-0.02 (-0.30, 0.26)	0.88
Kikuyu	0.64 (0.34, 1.19)	0.16	0.35 (0.17, 0.72)	0.0042	0.34 (0.21, 0.57)	<0.0001	0.25 (-0.14, 0.63)	0.21	0.55 (0.33, 0.76)	<0.0001	0.50 (0.22, 0.78)	0.00044
Kisii	0.69 (0.36, 1.30)	0.25	0.40 (0.19, 0.87)	0.021	0.48 (0.28, 0.82)	0.0068	0.22 (-0.17, 0.62)	0.27	0.49 (0.27, 0.72)	<0.0001	0.47 (0.18, 0.76)	0.0013
Lomwe	1.29 (0.80, 2.08)	0.30	0.35 (0.19, 0.64)	0.00075	0.43 (0.28, 0.65)	<0.0001	-0.11 (-0.41, 0.19)	0.49	0.66 (0.49, 0.84)	<0.0001	0.38 (0.16, 0.60)	0.00065
Luhya	0.72 (0.39, 1.35)	0.31	0.30 (0.14, 0.62)	0.0011	0.37 (0.22, 0.62)	0.00016	0.22 (-0.17, 0.61)	0.26	0.60 (0.39, 0.81)	<0.0001	0.54 (0.26, 0.82)	0.00014
Luo	0.67 (0.36, 1.25)	0.21	0.30 (0.14, 0.61)	0.0010	0.34 (0.21, 0.57)	<0.0001	0.37 (-0.02, 0.76)	0.064	0.61 (0.40, 0.82)	<0.0001	0.64 (0.36, 0.92)	<0.0001
Mandinka	0.96 (0.88, 1.05)	0.37	0.87 (0.76, 0.99)	0.034	0.88 (0.80, 0.98)	0.017	0.00 (-0.06, 0.07)	0.90	0.10 (0.04, 0.15)	0.00042	0.06 (0.02, 0.11)	0.0070
Mende	1.01 (0.80, 1.27)	0.96	1.16 (0.81, 1.68)	0.42	0.96 (0.73, 1.26)	0.77	-0.12 (-0.28, 0.05)	0.17	0.15 (0.02, 0.28)	0.028	0.01 (-0.12, 0.13)	0.91
Mijikenda	0.95 (0.51, 1.79)	0.88	0.51 (0.25, 1.06)	0.071	0.60 (0.36, 1.01)	0.054	0.03 (-0.37, 0.43)	0.88	0.35 (0.13, 0.57)	0.0020	0.24 (-0.04, 0.53)	0.095
Mole-Dagbani	0.57 (0.30, 1.08)	0.085	0.74 (0.36, 1.52)	0.42	0.57 (0.34, 0.95)	0.033	0.34 (-0.06, 0.73)	0.093	0.12 (-0.10, 0.34)	0.28	0.25 (-0.03, 0.54)	0.081
Mossi	0.92 (0.76, 1.10)	0.35	0.82 (0.64, 1.04)	0.10	0.80 (0.66, 0.98)	0.030	0.07 (-0.08, 0.21)	0.36	0.08 (-0.04, 0.20)	0.18	0.10 (-0.01, 0.20)	0.072
Ngoni	1.36 (0.85, 2.17)	0.20	0.49 (0.28, 0.85)	0.012	0.48 (0.32, 0.71)	0.0003	-0.16 (-0.46, 0.13)	0.28	0.74 (0.57, 0.91)	<0.0001	0.40 (0.19, 0.61)	0.00023
Sara	0.43 (0.29, 0.64)	<0.0001	0.42 (0.25, 0.69)	0.00072	0.41 (0.27, 0.61)	<0.0001	0.44 (0.15, 0.72)	0.0027	0.59 (0.38, 0.81)	<0.0001	0.73 (0.51, 0.94)	<0.0001
Senoufo	1.16 (1.00, 1.34)	0.053	0.92 (0.75, 1.13)	0.41	1.00 (0.85, 1.18)	0.96	-0.16 (-0.27, -0.05)	0.0054	0.08 (-0.02, 0.17)	0.10	-0.04 (-0.12, 0.04)	0.33

Table S3. Association between ethnicity and growth outcomes in children (n=138,312) aged <5 years in sub-Saharan Africa

	**Stunting**		**Wasting**		**Underweight**		**HAZ score**		**WHZ score**		**WAZ score**	
	OR (95% CI)	p	OR (95% CI)	p	OR (95% CI)	p	Coefficient (95% CI)	p	Coefficient (95% CI)	p	Coefficient (95% CI)	p
Serere	0.84 (0.75, 0.95)	0.0054	0.67 (0.56, 0.80)	<0.0001	0.67 (0.59, 0.77)	<0.0001	0.08 (-0.00, 0.15)	0.054	0.11 (0.04, 0.18)	0.0011	0.11 (0.06, 0.17)	0.00013
Soninke/Serahuleh	0.90 (0.79, 1.04)	0.16	0.89 (0.74, 1.07)	0.23	0.81 (0.69, 0.94)	0.0059	0.00 (-0.10, 0.10)	0.98	0.10 (0.01, 0.18)	0.024	0.07 (-0.00, 0.14)	0.063
Temne	0.92 (0.72, 1.17)	0.50	1.07 (0.73, 1.56)	0.72	0.99 (0.75, 1.31)	0.93	-0.02 (-0.19, 0.15)	0.82	0.04 (-0.10, 0.18)	0.57	-0.00 (-0.13, 0.13)	0.97
Tiv	0.46 (0.38, 0.56)	<0.0001	0.45 (0.34, 0.61)	<0.0001	0.31 (0.24, 0.39)	<0.0001	0.50 (0.35, 0.65)	<0.0001	0.61 (0.48, 0.74)	<0.0001	0.72 (0.60, 0.83)	<0.0001
Tonga	1.11 (0.70, 1.78)	0.65	0.25 (0.14, 0.44)	<0.0001	0.44 (0.29, 0.65)	<0.0001	-0.16 (-0.45, 0.14)	0.30	0.63 (0.46, 0.80)	<0.0001	0.35 (0.13, 0.56)	0.0013
Tumbuka	1.32 (0.82, 2.11)	0.25	0.29 (0.16, 0.51)	<0.0001	0.44 (0.29, 0.65)	<0.0001	-0.24 (-0.53, 0.06)	0.12	0.78 (0.61, 0.95)	<0.0001	0.40 (0.19, 0.61)	0.00025
Ubangi and Itimbiri	1.09 (0.58, 2.07)	0.79	0.86 (0.41, 1.81)	0.70	0.73 (0.43, 1.25)	0.25	0.02 (-0.38, 0.43)	0.91	0.30 (0.07, 0.54)	0.011	0.21 (-0.08, 0.50)	0.16
Wollof	0.87 (0.80, 0.95)	0.0013	0.85 (0.76, 0.96)	0.01	0.78 (0.71, 0.86)	<0.0001	0.06 (0.00, 0.12)	0.039	0.07 (0.02, 0.12)	0.0056	0.07 (0.03, 0.11)	0.0010
Xichangana	0.72 (0.30, 1.71)	0.46	0.19 (0.07, 0.51)	0.0012	0.29 (0.15, 0.60)	0.0007	0.12 (-0.42, 0.66)	0.66	0.92 (0.63, 1.21)	<0.0001	0.68 (0.30, 1.06)	0.00052
Yoruba	0.58 (0.53, 0.65)	<0.0001	0.65 (0.56, 0.75)	<0.0001	0.53 (0.47, 0.60)	<0.0001	0.37 (0.29, 0.45)	<0.0001	0.26 (0.20, 0.33)	<0.0001	0.37 (0.31, 0.43)	<0.0001
Covariables												
Accessibility to cities	1.03 (0.99, 1.07)	0.12	1.04 (0.99, 1.10)	0.16	1.06 (1.02, 1.10)	0.006	0.00 (-0.02, 0.03)	0.79	-0.03 (-0.05, -0.01)	0.011	-0.02 (-0.04, -0.00)	0.030
Age in days	1.00 (1.00, 1.00)	<0.0001	1.00 (1.00, 1.00)	<0.0001	1.00 (1.00, 1.00)	<0.0001	-0.00 (-0.00, -0.00)	<0.0001	-0.00 (-0.00, -0.00)	<0.0001	-0.00 (-0.00, -0.00)	<0.0001
Caregiver secondary education	0.87 (0.84, 0.89)	<0.0001	0.92 (0.87, 0.97)	0.0024	0.88 (0.84, 0.91)	<0.0001	0.11 (0.09, 0.14)	<0.0001	0.03 (0.01, 0.05)	0.0023	0.08 (0.07, 0.10)	<0.0001
DPT-3 vaccination	0.95 (0.92, 0.99)	0.0060	0.98 (0.93, 1.04)	0.56	0.96 (0.92, 1.00)	0.078	-0.00 (-0.03, 0.02)	0.75	-0.06 (-0.08, -0.04)	<0.0001	-0.04 (-0.06, -0.02)	<0.0001
Diarrhoea in past 2 weeks	1.24 (1.20, 1.28)	<0.0001	1.27 (1.21, 1.34)	<0.0001	1.44 (1.39, 1.50)	<0.0001	-0.21 (-0.23, -0.18)	<0.0001	-0.19 (-0.21, -0.17)	<0.0001	-0.24 (-0.26, -0.22)	<0.0001
Finished floor material	0.86 (0.83, 0.89)	<0.0001	1.00 (0.95, 1.05)	0.92	0.90 (0.87, 0.94)	<0.0001	0.11 (0.08, 0.13)	<0.0001	-0.01 (-0.03, 0.01)	0.37	0.06 (0.04, 0.07)	<0.0001
Household wealth index	0.69 (0.67, 0.72)	<0.0001	0.76 (0.72, 0.81)	<0.0001	0.66 (0.64, 0.69)	<0.0001	0.26 (0.24, 0.28)	<0.0001	0.12 (0.10, 0.14)	<0.0001	0.23 (0.21, 0.25)	<0.0001
ITN use night before	0.94 (0.91, 0.96)	<0.0001	0.97 (0.93, 1.02)	0.21	0.95 (0.92, 0.98)	0.0036	0.03 (0.01, 0.05)	0.0091	0.00 (-0.02, 0.02)	0.85	0.02 (0.00, 0.03)	0.02
Improved sanitation	0.92 (0.89, 0.95)	<0.0001	0.98 (0.93, 1.03)	0.40	0.94 (0.91, 0.98)	0.0018	0.07 (0.04, 0.09)	<0.0001	0.02 (0.00, 0.04)	0.045	0.05 (0.03, 0.07)	<0.0001
Improved water	0.95 (0.93, 0.98)	0.0023	1.00 (0.95, 1.04)	0.87	0.97 (0.94, 1.00)	0.085	0.02 (-0.00, 0.04)	0.064	-0.01 (-0.03, 0.01)	0.41	0.01 (-0.01, 0.02)	0.52
LST ≥35ºC	0.95 (0.91, 1.00)	0.034	1.12 (1.05, 1.20)	0.00092	1.08 (1.03, 1.14)	0.0041	0.01 (-0.02, 0.05)	0.45	-0.13 (-0.16, -0.10)	<0.0001	-0.08 (-0.11, -0.05)	<0.0001
Male	1.30 (1.27, 1.33)	<0.0001	1.21 (1.17, 1.26)	<0.0001	1.24 (1.21, 1.28)	<0.0001	-0.19 (-0.20, -0.17)	<0.0001	-0.04 (-0.06, -0.03)	<0.0001	-0.11 (-0.12, -0.10)	<0.0001
Measles vaccination	1.29 (1.25, 1.34)	<0.0001	1.01 (0.96, 1.07)	0.68	1.07 (1.03, 1.12)	0.00063	-0.18 (-0.21, -0.16)	<0.0001	-0.05 (-0.07, -0.03)	<0.0001	-0.04 (-0.06, -0.03)	<0.0001
Measurement position	0.85 (0.82, 0.88)	<0.0001	0.69 (0.65, 0.73)	<0.0001	-	-	0.23 (0.21, 0.26)	<0.0001	0.23 (0.21, 0.25)	<0.0001	-	-
Rainfall	0.97 (0.90, 1.05)	0.48	0.77 (0.68, 0.87)	<0.0001	0.81 (0.74, 0.89)	<0.0001	0.05 (0.00, 0.11)	0.040	0.08 (0.03, 0.12)	0.00059	0.08 (0.04, 0.12)	0.00015
Urban cluster	0.86 (0.82, 0.89)	<0.0001	1.03 (0.97, 1.10)	0.32	0.89 (0.85, 0.94)	<0.0001	0.15 (0.12, 0.18)	<0.0001	0.00 (-0.03, 0.03)	0.98	0.09 (0.06, 0.11)	<0.0001
Vegetation index	1.06 (1.01, 1.11)	0.016	0.84 (0.77, 0.90)	<0.0001	0.96 (0.91, 1.02)	0.24	-0.07 (-0.10, -0.03)	0.00012	0.07 (0.04, 0.10)	<0.0001	0.01 (-0.02, 0.04)	0.41

CI: Confidence intervals; HAZ: height-for-age z-score; LST: land surface temperature OR: Odds Ratio; WAZ: weight-for-age z-score; WHZ: weight-for-height z-score.
