# Supplementary material for: Ethnicity and anthropometric deficits in children: A cross-sectional analysis of national survey data from 18 countries in sub-Saharan Africa
Source: PLOS Glob Public Health. 2024 Dec 31;4(12):e0003067. doi: 10.1371/journal.pgph.0003067 (PMC11687787; doi:10.1371/journal.pgph.0003067)
Supplement: S1 Fig — Figures show significant pair-wise comparisons of mean weight-for-height z-scores and weight-for-age z-scores between ethnic groups. (PDF) [file pgph.0003067.s006.pdf]

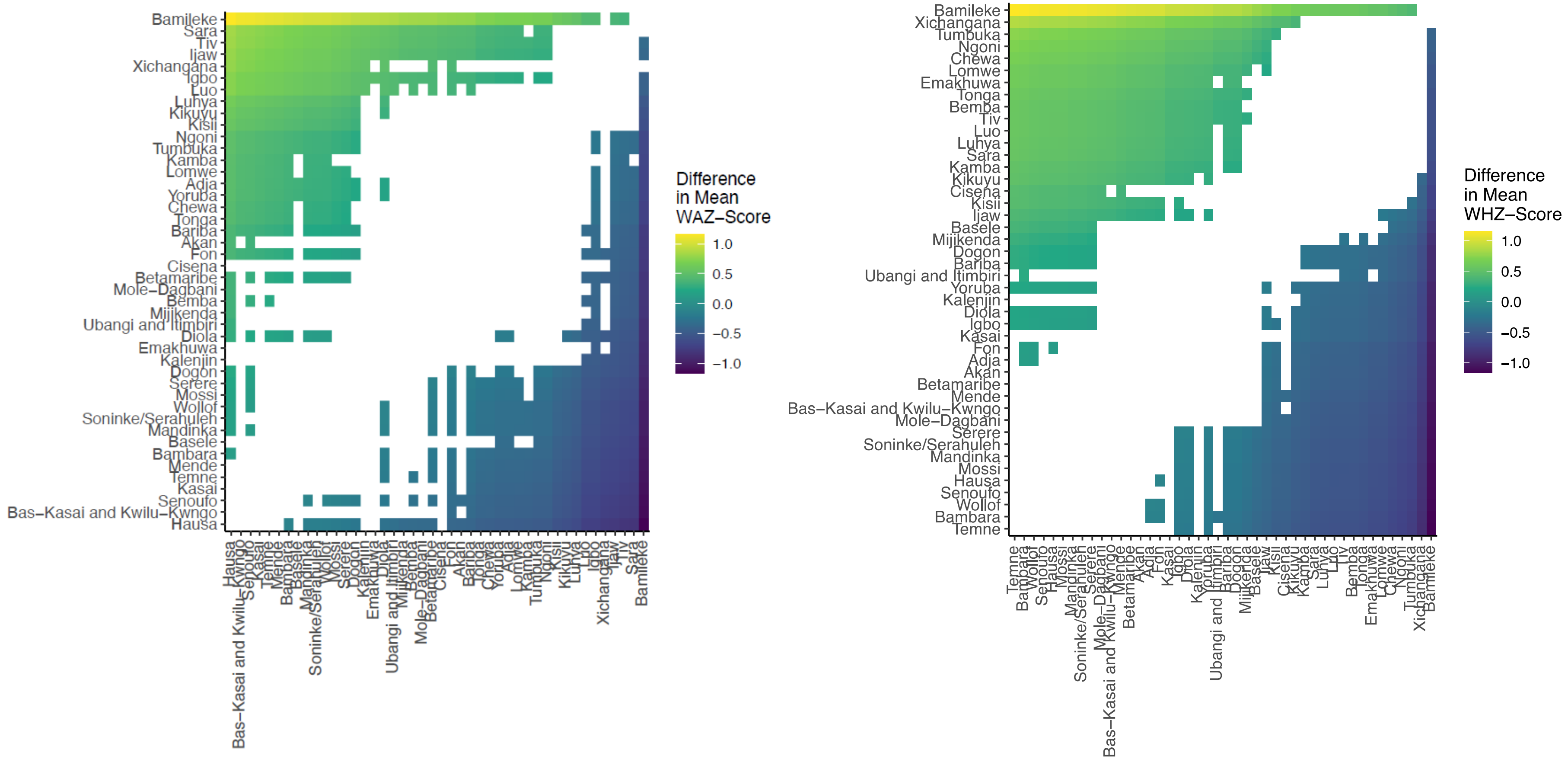

Figure S1. Growth variation relative to ethnic group among 138,312 children aged <5 years in 18 countries in Sub-Saharan Africa surveyed between 2006 to 2019. Figures show significant pair-wise comparisons of mean weight-for-age *z* (WAZ) scores and weight-for-height *z* (WHZ) scores between ethnic groups.
