# Supplementary material for: Ethnicity and anthropometric deficits in children: A cross-sectional analysis of national survey data from 18 countries in sub-Saharan Africa
Source: PLOS Glob Public Health. 2024 Dec 31;4(12):e0003067. doi: 10.1371/journal.pgph.0003067 (PMC11687787; doi:10.1371/journal.pgph.0003067)
Supplement: S2 Fig — AUC: Area under curve; cor: correlation. (PDF) [file pgph.0003067.s007.pdf]

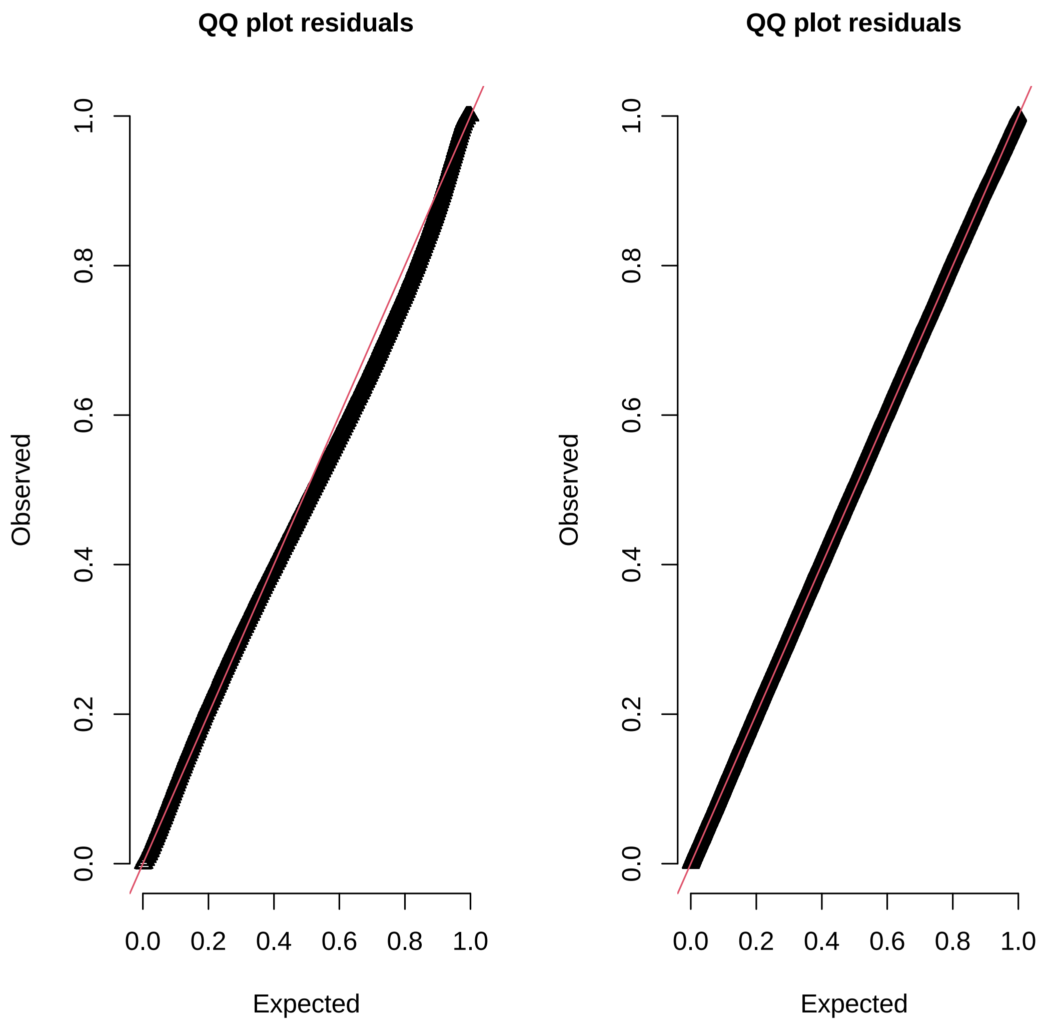

Figure S2. Model validation for height-for-age z-score. Calibration plots for the Gaussian and Binomial models. AUC for individual based predictions 0.74
